# Digital Holographic Microscopy for Non-Invasive Monitoring of Cell Cycle Arrest in L929 Cells

**DOI:** 10.1371/journal.pone.0106546

**Published:** 2014-09-10

**Authors:** Maria Falck Miniotis, Anthonny Mukwaya, Anette Gjörloff Wingren

**Affiliations:** Department of Biomedical Laboratory Science, Health and Society, Malmö University and Malmö University Hospital, Malmö, Sweden; Dalhousie University, Canada

## Abstract

Digital holographic microscopy (DHM) has emerged as a powerful non-invasive tool for cell analysis. It has the capacity to analyse multiple parameters simultaneously, such as cell- number, confluence and phase volume. This is done while cells are still adhered and growing in their culture flask. The aim of this study was to investigate whether DHM was able to monitor drug-induced cell cycle arrest in cultured cells and thus provide a non-disruptive alternative to flow cytometry. DHM parameters from G1 and G2/M cell cycle arrested L929 mouse fibroblast cells were collected. Cell cycle arrest was verified with flow cytometry. This study shows that DHM is able to monitor phase volume changes corresponding to either a G1 or G2/M cell cycle arrest. G1-phase arrest with staurosporine correlated with a decrease in the average cell phase volume and G2/M-phase arrest with colcemid and etoposide correlated with an increase in the average cell phase volume. Importantly, DHM analysis of average cell phase volume was of comparable accuracy to flow cytometric measurement of cell cycle phase distribution as recorded following dose-dependent treatment with etoposide. Average cell phase volume changes in response to treatment with cell cycle arresting compounds could therefore be used as a DHM marker for monitoring cell cycle arrest in cultured mammalian cells.

## Introduction

On-going developments in the field of cancer therapeutics are increasingly directed towards personalised medicine with a focus on target based drugs. Such compounds are often aimed against specific pathways that are frequently deregulated in cancer [1] including those that stimulate cell proliferation by enabling unhindered cell division [2]. In fact, the majority of the proliferation-associated genes are cell cycle regulated [3]. Compared to more conventional cytotoxic therapy, many of these emerging targeted anticancer drugs are therefore inherently cytostatic and cause cell cycle arrest.

Cell cycle monitoring can be exploited for evaluating drug action. This is important because alongside the development of more efficacious, targeted treatments, it is equally crucial to tailor each treatment individually, at an early stage. Monitoring drug effect can help avoid the cancer from spreading and/or developing drug resistance as a result of an ineffective treatment approach.

Flow cytometry analysis of cell cycle profiles is often employed for *ex vivo/in vitro* information on drug action. The key benefit of this approach is a direct assay of cell cycle profiles as detection relies on DNA staining. The amount of DNA intercalation is correlated to the different stages of the cell cycle as the cell generates duplicate DNA before cell division. However, this method requires removal of a portion of the cancer cells from their culture environment or wasting precious patient samples in order to label cells for analysis. Thus, this invasive multi-step approach is sample-wasting and time-consuming and calls for new and improved technology for cancer cell analysis of response to targeted treatment which is urgently needed in order to overcome these issues.

We propose the use of a low intensity laser imaging technique, digital holographic microscopy (DHM), for assessing drug induced cell cycle alterations. DHM, which has recently increased in popularity, is a high-resolution imaging technique that enables real-time detection and quantification of both single as well as whole populations of cells, without the need for prior cell extraction, staining or exposing cells to harmful light sources. Compared to conventional approaches, DHM allows non-destructive characterization of cell- number, confluence, shape, phase volume etc. of which can be related to cell proliferation and apoptosis [4].

Kemper and colleagues have recently measured the length of the cell cycle of an individual cell using DHM [5]. By using DHM, we have studied the morphology of an individual pancreatic cancer cell undergoing cell division [4]. As yet, the technique has not been developed to perform actual cell cycle studies. Here, we evaluated the feasibility of DHM for monitoring cell cycle alterations induced by cell cycle arresting compounds. The aim was to exploit the capacity of DHM to identify specific changes in cell phase volume that correlate to either a G1 or a G2/M arrest.

By monitoring changes in cell phase volume, we hypothesize that DHM will be able to detect an accumulation of cells in either the G1 or the G2/M cell cycle phase by utilizing the fact that G1 cells are smaller than G2/M cells. To test this hypothesis, G1 and G2/M cell cycle arrest was induced in L929 mouse fibroblast cells by three different compounds. In order to identify doses that achieved cell cycle arrest, flow cytometry was applied. Doses that successfully arrested cells were used for further DHM studies. DHM images were acquired on live cells and information on cell- number, confluence and phase volume were collected where after cells were harvested for continuous verification of cell cycle arrest (by flow cytometric acquisition).

Protein kinase inhibitor staurosporine, a known G1 arresting compound was used to induce G1 arrest [6]. Additionally, anti-tubulin drug colcemid [7] and anti-cancer substance etoposide, known to induce a strong G2/M arrest [8], [9], was used for this purpose. Furthermore, etoposide was used in a dose-dependent manner in order to compare the sensitivity of DHM to flow cytometry for detecting drug-induced cell cycle arrest.

Our data presented herein agrees with the overall aim of this study, to investigate whether DHM is able to monitor drug-induced cell cycle arrest in cultured cells and thus provide a non-disruptive alternative to flow cytometry.

## Materials and Methods

### Routine culture

L929 mouse fibroblast cells (Invitrogen) were grown as a monolayer in minimum eagle’s media (Invitrogen) supplemented with 10% fetal calf serum (FCS, Invitrogen) and 1% penicillin/streptomycin. Cells were kept in a humidified incubator at 37°C with 5% CO_2_. Growth media was replenished every 48 h and cells were re-seeded once a week. To avoid genetic drift and in order to minimise the risk of contamination, cells were not cultured for longer than two months at a time.

### Cell treatments

Cells were seeded at 2.5×10^3^ cells/flask in 25 cm^2^ polystyrene tissue culture vessels containing 4.3 ml of media without FCS. 24 h later, cells were treated with 20 nM staurosporine (Invitrogen) or 3 µM colcemid (Santa Cruz) or 0.1–10 µM etoposide (Sigma-Aldrich) or with a 0.1% concentration of drug solvent dimethyl sulfoxide (DMSO, Sigma-Aldrich) in media with FCS. 24 h post-treatment DHM analysis (see description below) was performed and cells were harvested with trypsin. Cell counts and viability was determined by standard trypan blue exclusion of dead cells prior to flow cytometric analysis (see description below).

### Digital holographic microscopy

Twenty 3D holographic cell images were captured using a HoloMonitor M3 (Phase Holographic Imaging AB) with a 20x magnification objective and equipped with a 0.8 mW HeNe laser (633 nm, 10 W/m^2^, exposure time <5 ms). Images were converted from wavelength interactions to cell representations by a computer algorithm (HstudioM3, Phase Holographic Imaging AB). From these computations, information on average cell number, confluence and cell phase volume could be obtained. Images were acquired in a short time-frame, within three minutes.

The cell phase volume, V_φ_ was calculated from eq. 1 [10]. S_c_ is the projected cell area on the x-y plane and OPD_c_ is the averaged OPD over the cell area.
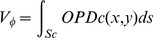
(1)


### Flow cytometry

1×10^5^ cells per sample were fixed on ice for 30 m with 70% ethanol (in phosphate buffered saline, PBS). Fixed cells were incubated for 30 m at 37°C in PBS with 10 µg/ml propidium iodide containing RNase (both from Sigma Aldrich), 0.1 mg/ml. A minimum of 3×10^3^ cells per sample were processed in an Accuri C6 (BD Biosciences) and analysed with associated software, in order to determine cell cycle phase distribution.

### MTS assay

CellTiter 96 Aqueous Non-Radioactive Cell Proliferation (MTS) assay was used according to suppliers’ instructions (Promega).

### Statistical analysis

Experiments were repeated on separate days. Data is expressed as mean ± standard deviation (STDEV). Statistical significance was determined by two-tailed unpaired Student’s t-test with P values ≤0.05 considered to be significant.

## Results

**Figure 1:**
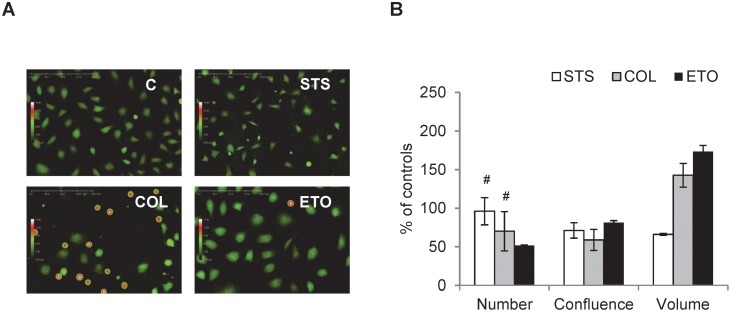
Digital holographic microscopy of staurosporine induced G1 arrest and etoposide or colcemid induced G2/M arrest. L929 cells treated with 20 nM staurosporine (STS) or 3 µM colcemid (COL) or 1 µM etoposide (ETO) for 24 h (untreated controls, C). **A.** Digital holographic microscopy images, artificially coloured, with increasing thickness shown as green<red<white. **B.** STS treatment has no significant effect on average cell numbers; however it decreases average confluence and average cell volume. COL treatment has no significant effect on average cell number, instead average cell confluence decreases and average cell volume increases. ETO treatment significantly lowers average cell numbers, average confluence and increases average cell volume. Images and histograms are representative of n3. All results are significant except # which indicates P>0.05 compared to controls.

DHM cell signatures were analysed post- staurosporine, colcemid or etoposide treatment, Fig. 1. The number of cells decreased regardless of treatment-type. In response to staurosporine treatment, the cells decreased in cell size, while in response to colcemid or etoposide treatment, the cell size increased, Fig. 1A. Colcemid treatment resulted in a mixed population of both larger and smaller sized cells, as compared to control cells. The effect on cell numbers post-treatment was significantly decreased only in response to etoposide treatment (as compared to controls). However, all treatments reduced cell confluence. Compared to controls, the cell phase volume decreased to 70% or increased to 140% and 170% for staruosporine, colcemid and etoposide treated cells respectively, Fig. 1B.

**Figure 2:**
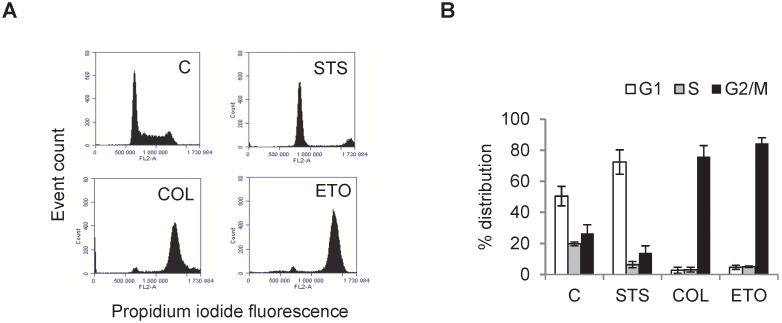
Flow cytometric measurement of staurosporine induced G1 arrest and colcemid or etoposide induced G2/M arrest. L929 cells treated with 20 nM staurosporine (STS) or 3 µM colcemid (COL) or 1 µM etoposide (ETO) for 24 h (untreated controls, C). **A.** DNA histograms obtained from flow cytometry measurements show a shift in the G2/M to G1 cell cycle phase for STS treatment and reversed for COL or ETO, a G1 to G2/M shift. **B.** STS increases G1 phase population of cells and COL and ETO increases G2/M phase population of cells. Images and histograms are representative of n3. All results are significant, P≤0.05 compared to controls.

Successful induction of cell cycle arrest following 24 h of treatment was achieved, Fig. 2. Flow cytometric histograms show untreated control cells and cells treated with staurosporine, colcemid or etoposide, Fig. 2A. Compared to untreated controls, the amount of cells in the S and G2/M phase decreased, while the amount of cells in the G1 phase accumulated in response to staurosporine treatment. In contrary to this, the number of cells in the G1 and S phase decreased and the number of cells in the G2/M phase increased post-treatment with colcemid or etoposide, Fig. 2B.

**Figure 3:**
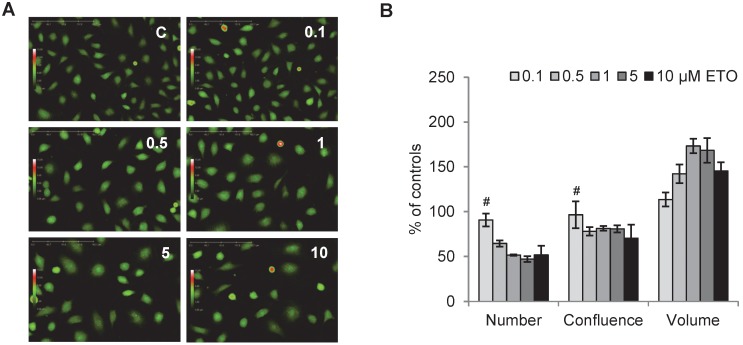
Digital holographic microscopy of dose-dependent etoposide induced G2/M arrest. L929 cells treated with 0.1–10 µM etoposide (ETO) for 24 h (untreated controls, C). **A.** Digital holographic microscopy images of a dose-dependent increase in the average cell volume. Images are artificially coloured, with increasing thickness shown as green<red<white. **B.** ETO reduces average cell number, decreases average cell confluence and increases average cell volume. Images and histograms are representative of n3. All results are significant except # which indicates P>0.05 compared to controls.

To summarise, DHM was able to detect changes in response to both a G1 or a G2/M cell cycle arrest in L929 cells. Etoposide was further used in a dose-dependent manner in order to investigate how well DHM is able to record a shift in the cell cycle profile, as compared to flow cytometry. Prior to flow cytometry processing and analysis, DHM images were acquired as shown in Fig. 3. With an increasing dose of etoposide, the number of cells decreased, accompanied by an increased cell size, Fig. 3A. As compared to untreated controls, the confluence and the number of cells left post-treatment was significantly decreased at all but the lowest dose, Fig. 3B. Fewer albeit larger cells could be observed at higher doses (Fig. 3A), reflecting the small changes on confluence. The cell phase volume peaked at 1 µM, as compared to untreated controls. At 10 µM, the cell phase volume decreased as compared to doses of 1–5 µM.

**Figure 4:**
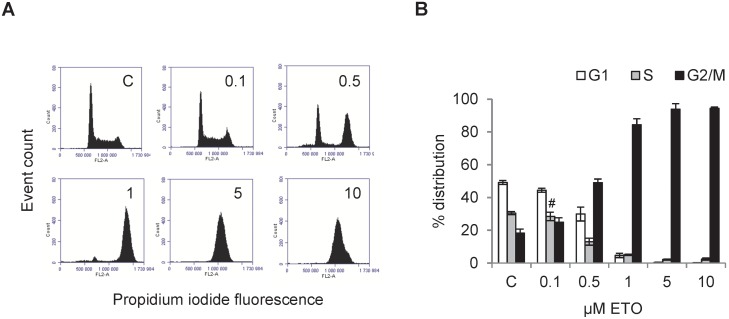
Flow cytometric measurement of dose-dependent etoposide induced G2/M arrest. L929 cells treated with 0.1–10 µM etoposide (ETO) for 24 h (untreated controls, C). **A.** DNA histograms obtained from flow cytometry measurements show a dose-dependent shift in the G1 to G2/M cell cycle phase. **B.** ETO increases G2/M phase population of cells and decreases S and G1 population of cells. Images and histograms are representative of n3. All results are significant except # which indicates P>0.05 compared to controls.

Dose-dependent G2/M cell cycle arrest was achieved by etoposide, Fig. 4. Flow cytometry histograms show untreated control cells and cells treated with an increasing dose of etoposide, Fig. 4A. The full dynamics of etoposide cell cycle arrest in L929 cells is shown with five different doses. At 0.1 µM, no effect could be observed as compared to control, however at 0.5 µM the distribution of cells start to shift from the G1 and S towards the G2/M phase. At 1 µM, only a few cells are found in the G1 and S phase since cells are arrested in the G2/M phase. All cells accumulated in the G2/M phase at 5 µM and increasing the dose to 10 µM does not improve this effect. In a dose-dependent manner, the number of G2/M phase cells increased and the number of G1 and S phase cells decreased significantly post-treatment (except S phase cells at the lowest dose of etoposide), Fig. 4B. Correlating well with the histogram in Fig. 4A, the maximum amount of cells in the G2/M phase is reached at 5 µM.

**Figure 5:**
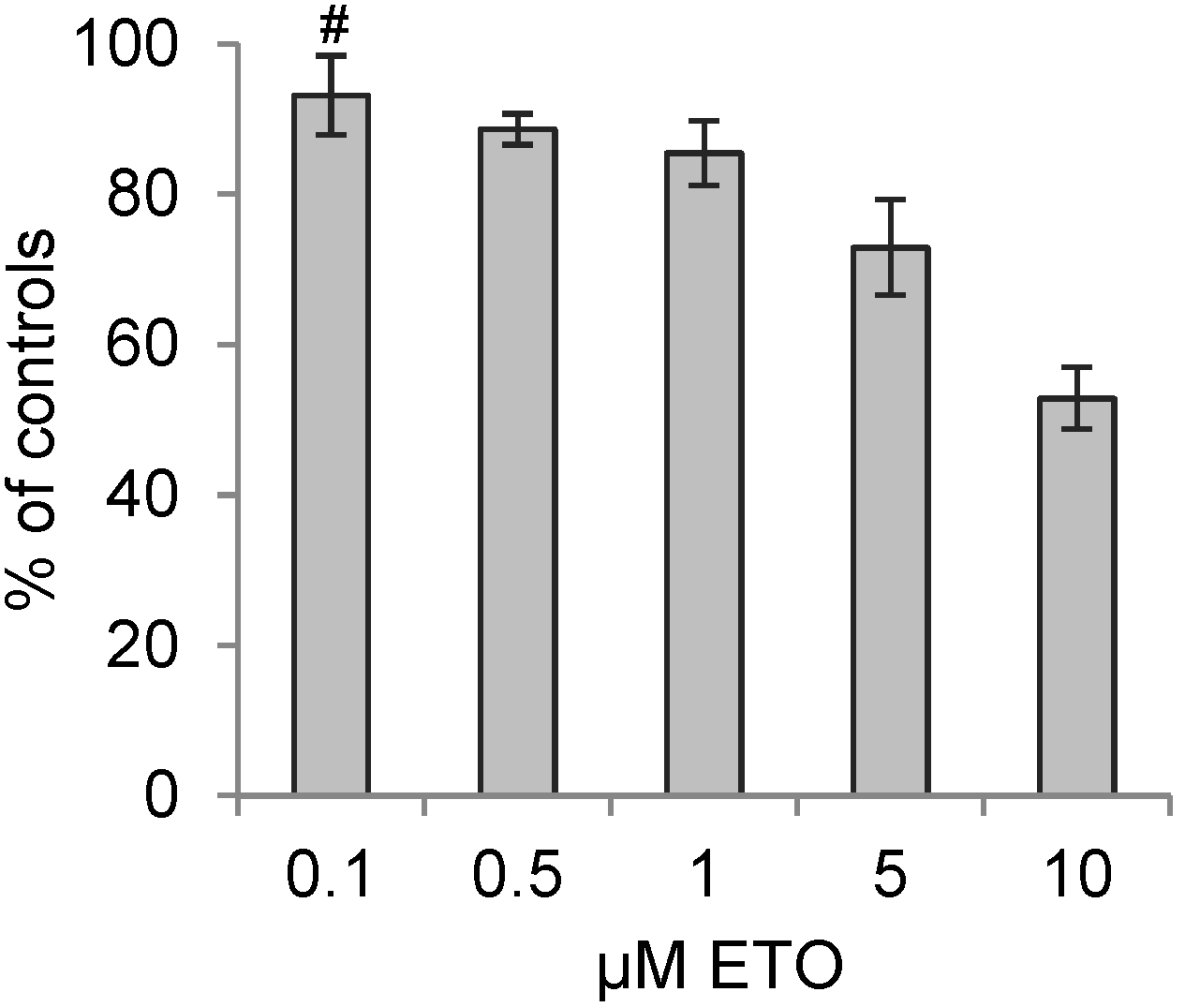
MTS cell viability measurement of dose-dependent etoposide induced G2/M arrest. L929 cells treated with 0.1–10 µM etoposide (ETO) for 24 h. **A.** MTS analysis show a dose-dependent shift in cell viability post-etoposide treatment as compared to controls. All results are significant except # which indicates P>0.05 compared to controls, n3.

Cell viability post-etoposide treatment was evaluated using MTS assay which measures the level of reductase activity. With increasing doses of etoposide, cell viability was reduced (Fig. 5). In addition, MTS readings showed higher viability after treatment with 1 µM etoposide as compared to 3 µM colcemid (85±4% vs 59±2% viable cells, p = 0.005).

In summary, at an etoposide dose where flow cytometry was yet unable to detect a significant G2/M arrest, DHM recorded a significant decrease in the phase volume. Moreover, while the number of cells in the G2/M phase remained relatively constant even at higher doses of etoposide, cell viability and the average phase volume decreased at doses above and beyond concentrations capable of inducing a G2/M arrest.

## Discussion

Over the last decade, DHM has emerged as a non-invasive alternative to traditional microscopy for studies on cultured cells. The technique has the potential to develop into a fast and cost-efficient method for pre-clinical monitoring of cancer treatment efficacy [4]. The aim of this study was to evaluate the potential of using DHM for analysis of cell cycle arrest, a common effect of many cytostatic cancer treatments [11].

The present study is the first to use DHM for monitoring drug induced cell cycle arrest. Moreover, rather than following individual cells over time, several cell populations are analysed at a given time point and average representations of cell parameters such as cell number, confluence and phase volume are presented. While flow cytometry is a direct representation of distribution of cells in various cell cycle phases; DHM could be used for indirect analysis of cell cycle arrest. Hence, for DHM, there is a need for simultaneous monitoring of additional cellular processes that can cause a change in cell phase volume. Despite this, we show that average cell phase volume results from DHM readings are comparable to the flow cytometry findings. In agreement with our hypothesis, staurosporine induced G1 arrest correlates with a significant decrease in cell phase volume whereas a colcemid or etoposide induced G2/M arrest correlate with a significant increase in cell phase volume.

The majority of DHM studies of mammalian cells to date have focused on cell death and changes in cell phase volume could be one way to determine onset of cell fragmentation [4], [12]–[14]. The effect on cell phase volume could partly reflect the effect of a G2/M arrest and partly the result of cell death. However, treatment with stauroporine does not affect cell numbers and hence does not imply that the dose is toxic to the cells. Instead, decreased cell confluence post-treatment with stauropsorine likely reflect a reduction in cell phase volume, specifically due to G1 arrest and not cell death. MTS evaluation showed higher cell viability after treatment with 1 µM etoposide as compared to 3 µM colcemid. This corresponds well with the observation of more apparently shrinking/dying cells and overall lower effect on cell phase volume in response to colcemid treatment. Staurosporine affects reductase activity and was therefore not included in the MTS analysis.

Etoposide was tested in a dose-dependent manner to be able to correlate flow cytometry findings to DHM measurements on average cell phase volume. The average cell phase volume increased more with etoposide than with any other compound used in this study, which is also in line with the ability of etoposide to induce the strongest G2/M arrest. A weak G2/M arrest could be detected already at 0.1 µM whereas the maximum G2/M arrest was observed at a dose between 1–5 µM. However, both 10 µM and 5 µM of etoposide show similar effects with flow cytometry. DHM measurements of average cell phase volume correlate well with the flow cytometry results. Already with a weak G2/M arrest, a significant increase in the average cell phase volume is observed. Similarly, phase volume max is reached at 1 µM etoposide, almost in line with flow cytometry findings where maximum cell cycle arrest is observed at 5 µM. These results show that DHM is a sensitive method for identifying cell cycle arrest.

The cell number goes down with increasing doses of etoposide and fewer albeit larger cells remain which explains the relatively constant effect on cell confluence. Cell phase volume is decreased at doses beyond maximum cell cycle arrest (5–10 µM), as compared to control (there is also a significant decrease between 1–10 µM, p = 0.02). MTS viability assay show a significant dose-dependent decrease in cell viability from 1 to 10 µM etoposide (p = 0.0002). The decreased cell phase volume at higher doses of etoposide is therefore likely due to dying cells.

This highlights that DHM is an indirect assay for cell cycle arrest, while flow cytometry gives a direct representation of the distribution of cells in the various cell cycle phases, as it relies of DNA staining. Nevertheless, if the correct dose is pre-evaluated, DHM has a clear advantage in terms of time-saving non-invasive monitoring and could be used for routine measurements of cell cycle arrest under defined conditions.

With flow cytometry, cells are measured one at a time. Depending on treatment- or cell type, cells can easily detach from the culture surface and when preparing samples for flow cytometry, many cells can be lost in the process. Cells that are still attached to the cell surface albeit loosely, are still included in the DHM analysis. Cells that have detached and are floating in the media are not in the same plane of view and are not included in the DHM readings. Moreover, some cell types are prone to form aggregates which make flow cytometric acquisition a challenge. This issue can be avoided when using DHM directly on cultured cells without prior detachment. DHM, however, requires special attention to avoiding confluence and making sure cells are grown well dispersed.

In addition to cell phase volume, confluence and number, multiple cell morphology parameters can be detected in real-time, such as eccentricity and roughness [4]. After analysis, the flask can be kept for future use, as shown in the present study which enabled the same flask to be used for both DHM and flow cytometric acquisition. Many DHM studies to date including our own have focused on adherent cell lines, however by using special culture chambers that align cells in a thin monolayer allows for analysis of suspension cells. Similarly, blood cancer cells or circulating tumour cells could be screened with DHM which in turn could offer an instant clinical value. Unlimited real-time data acquisition and the ability to read 96-well plates with DHM [14] open up the ability for high-throughput screening of cell cycle arresting compounds.

In the present study, we report cell cycle dependent phase volume changes. Importantly, phase volume measurements do not provide an explicit means to decouple refractive index and thickness, which may inhibit studies looking for specific mechanisms for the changes in cell cycle. It is likely that cell cycle arrest induces changes in chromatin density which would lead to refractive index changes. The overall refractive index decrease during neutrophil and monocyte differentiation, associated with changes in subnuclear heterochromatin [15]. Refractive index changes could be measured according to previously published methods. These include off-axis DHM [15], [16] or a decoupling procedure to separately measure refractive index and thickness from the quantitative phase images of living cells [17]. For more precise estimation of the biovolume, off-axis DHM tomography or interferometric chamber coupled with wide-field digital interferometry could be used [18], [19]. In addition, spatial light interference microscopy (SLIM), white light optical interferometry modality designed as an add-on for commercial phase contrast microscopy has been developed to measure cell size and cell dry mass [20]. Similar to DHM, SLIM offers the possibility for monitoring single cells as well as bulk population of cells. Even though these modalities are not currently available commercially, they could offer more advanced analysis of biovolume changes.

To date, only two previous studies have used DHM for recording cell division. One study presented time-laps of endothelial cells undergoing cell division [5] and another investigated cell division of osteoblasts under simulated zero gravity condition [21]. Using immortalised murine fibroblast cells, this first proof-of-concept study suggests DHM as a possible alternative tool for analysis of cell cycle alterations. The next stage would be to treat cancer cell lines with clinically relevant anti-cancer compounds, for instance cell signalling pathway inhibitors which induce G1 cell cycle arrest as a part of their mechanism of action. For some compounds, this arrest can be directly translated to treatment sensitivity in cell models [22] while the G1 arrest is reversed upon resistance to treatment [23]. In the future, DHM could provide a pre-clinical tool for monitoring such targeted signalling inhibitors to assess treatment sensitivity or induction of resistance. More work in this area will show the feasibility of DHM for non-invasive analysis of the effect of cytostatic cancer treatment and the ability to identify drug-responding vs. non-responding treatment-insensitive cells. This provides an important tool for the study of mechanistic drivers of cancer cell growth and in the development and evaluation of cytostatic cancer treatments.
